# Supplementary material for: Prognostic value of a modified pathological staging system for gastric cancer based on the number of retrieved lymph nodes and metastatic lymph node ratio
Source: PeerJ. 2024 Oct 1;12:e18165. doi: 10.7717/peerj.18165 (PMC11451444; doi:10.7717/peerj.18165)
Supplement: Supplemental Information 1 [file peerj-12-18165-s001.docx]

Supplementary Material

**
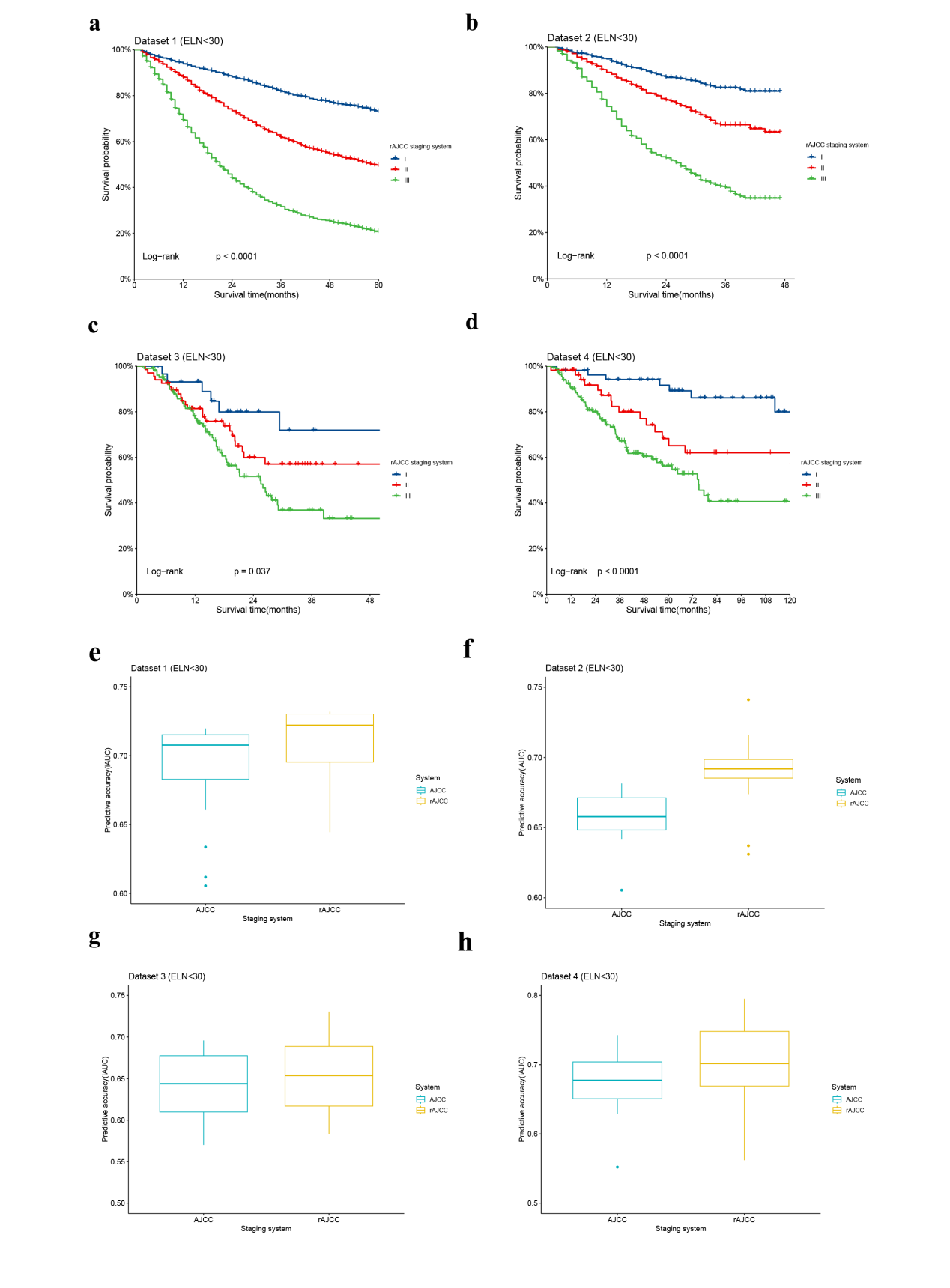
**

**Supplementary Figure 1.** The Kaplan-Meier survival curves for gastric cancer patients with ELN (Examined Lymph Nodes) less than 30 in the (a) Dataset 1, (b) Dataset 2, (c) Dataset 3 and (d) Dataset 4 were depicted according to the rAJCC staging system.

Performance of the rAJCC staging systems compared with the 8th AJCC staging system in the (e) Dataset 1, (f) Dataset 2, (g) Dataset 3 and (h) Dataset 4 with ELN less than 30.


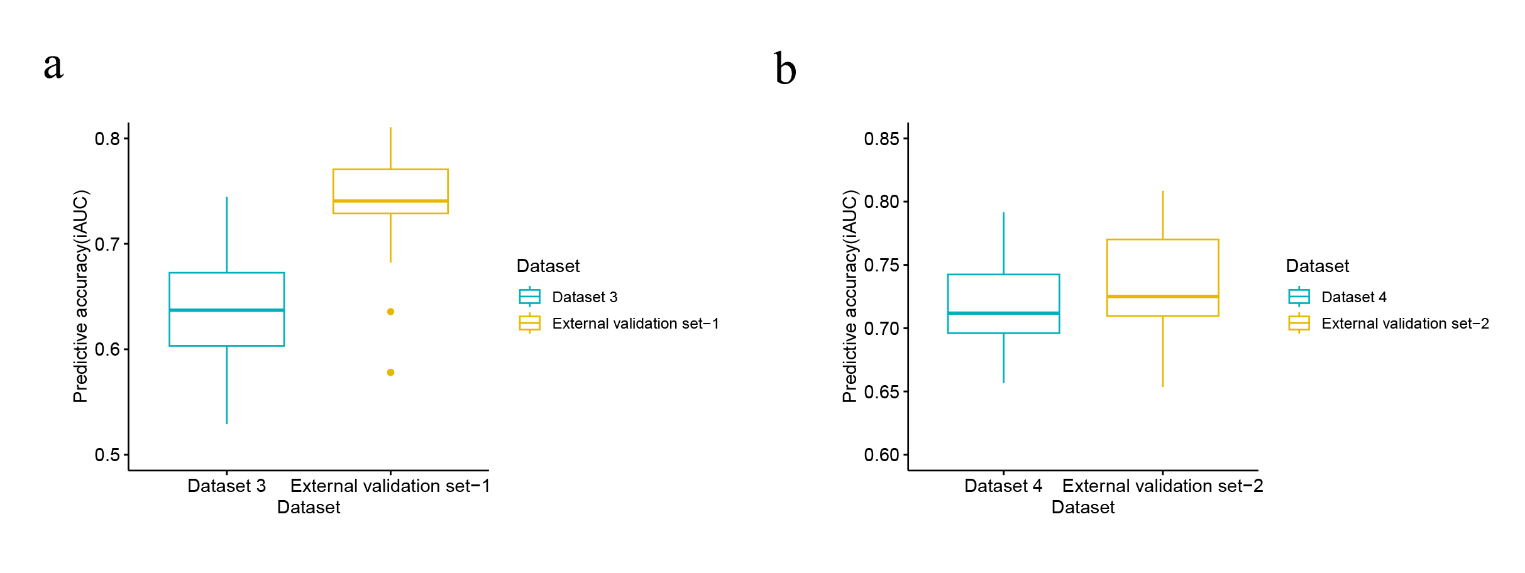


**Supplementary Figure 2.** Performance of the rAJCC staging systems incorporating ELN compared with the rAJCC staging system without ELN restriction in the a.TCGA dataset and b. Third Affiliated Hospital of Sun Yat-sen University dataset.

**Supplementary Table 1.** Demographic and pathological characteristics of study population.

| Characteristics | Dataset 1 | Dataset 2 | Dataset 3 | Dataset 4 |
| --- | --- | --- | --- | --- |
|  | N = 6351 (100%) | N=1786(100%) | N=277(100%) | N=719(100%) |
| Sex (%) |  |  |  |  |
| Male | 4242 (66.79) | 1206 (67.53) | 178 (64.26) | 486 (67.59) |
| Female | 2109 (33.21) | 580 (32.47) | 99 (35.74) | 233 (32.41) |
| Age (%) |  |  |  |  |
| <50 | 613 (9.65) | 176 (9.85) | 21 (7.58) | 149 (20.72) |
| 50-69 | 3067 (48.29) | 904 (50.62) | 148 (53.43) | 435 (60.50) |
| >69 | 2671 (42.06) | 706 (39.53) | 107 (38.63) | 135 (18.78) |
| Unknown | / | / | 1 (0.36) |  |
| Year (%) |  |  |  |  |
| 2010 | 1071 (16.86) | / | / | / |
| 2011 | 1046 (16.47) | / | / | / |
| 2012 | 1074 (16.91) | / | / | / |
| 2013 | 1049 (16.52) | / | / | / |
| 2014 | 1112 (17.51) | / | / | / |
| 2015 | 999 (15.73) | / | / | / |
| 2016 | / | 861 (48.21) | / | / |
| 2017 | / | 925 (51.79) | / | / |
| Race (%) |  |  |  |  |
| White | 4171 (65.67) | 1123 (62.88) | 189 (68.23) | / |
| Black | 756 (11.90) | 214 (11.98) | 7 (2.53) | / |
| Other | 1398 (22.01) | 431 (24.13) | 51 (18.41) | 719(100) |
| Unknown | 26 (0.41) | 18 (1.01) | 30 (10.83) | / |
| Grade (%) |  |  |  |  |
| I | 510 (8.03) | 141 (7.89) | 3 (1.08) | 18 (2.50) |
| II | 2054 (32.34) | 568 (31.80) | 94 (33.94) | 156 (21.70) |
| III | 3390 (53.38) | 935 (52.35) | 175 (63.18) | 494 (68.71) |
| IV | 107 (1.68) | 27 (1.51) | / | 51 (7.09) |
| Unknown | 290 (4.57) | 115 (6.44) | 5 (1.81) | / |
| Tumor size (%) |  |  |  |  |
| ＜5cm | 3591 (56.54) | 1012 (56.66) | 134 (48.38) | 453 (63.00) |
| ≥5cm | 2101 (33.08) | 495 (27.72) | / | 234 (32.55) |
| Unknown | 659 (10.38) | 279 (15.62) | 143 (51.62) | 32 (4.45) |
| Primary site (%) |  |  |  |  |
| Body | 594 (9.35) | 210 (11.76) | 72 (25.99) | 191 (26.56) |
| Antrum/pylorus | 1796 (28.28) | 501 (28.05) | 89 (32.13) | 289 (40.19) |
| Cardia/fundus | 2226 (35.05) | 596 (33.37) | 106 (38.27) | 145 (20.17) |
| Greater curvature | 255 (4.02) | 62 (3.47) | / | 3 (0.42) |
| Lesser curvature | 685 (10.79) | 182 (10.19) | / | 7 (0.97) |
| Overlapping regions | 400 (6.30) | 131 (7.33) | / | 42 (5.84) |
| Stomach NOS | 395 (6.22) | 104 (5.82) | 10 (3.61) | 42 (5.84) |
| 8th AJCC T stage (%) |  |  |  |  |
| T1 | 1633 (25.71) | 416 (23.29) | 18 (6.50) | 116 (16.13) |
| T2 | 940 (14.80) | 279 (15.62) | 67 (24.19) | 119 (16.55) |
| T3 | 2612 (41.13) | 771 (43.17) | 135 (48.74) | 88 (12.24) |
| T4a | 865 (13.62) | 259 (14.50) | 37 (13.36) | 294 (40.89) |
| T4b | 301 (4.74) | 61 (3.42) | 20 (7.22) | 102 (14.19) |
| 8th AJCC N stage (%) |  |  |  |  |
| N0 | 2761 (43.47) | 761 (42.61) | 85 (30.69) | 287 (39.92) |
| N1 | 1558 (24.53) | 444 (24.86) | 73 (26.35) | 110 (15.30) |
| N2 | 1044 (16.44) | 312 (17.47) | 64 (23.10) | 124 (17.25) |
| N3 | 988 (15.56) | 269 (15.06) | 55 (19.86) | 198 (27.54) |
| 8th AJCC TNM stage (%) |  |  |  |  |
| I | 1841 (28.99) | 488 (27.32) | 42 (15.16) | 175 (24.34) |
| II | 1817 (28.61) | 524 (29.34) | 96 (34.66) | 161 (22.39) |
| III | 2693 (42.40) | 774 (43.34) | 139 (50.18) | 383 (53.27) |
| rN stage (%) |  |  |  |  |
| rN0 | 3192 (50.26) | 960 (53.75) | 90 (32.49) | 306 (42.56) |
| rN1 | 1204 (18.96) | 335 (18.76) | 54 (19.49) | 196 (27.26) |
| rN2 | 1023 (16.11) | 265 (14.84) | 57 (20.58) | 139 (19.33) |
| rN3a | 328 (5.16) | 69 (3.86) | 25 (9.03) | 38 (5.29) |
| rN3b | 604 (9.51) | 157 (8.79) | 51 (18.41) | 40 (5.56) |
| rAJCC stage (%) |  |  |  |  |
| rI | 2093 (32.96) | 563 (31.52) | 43 (15.52) | 189 (26.29) |
| rII | 2234 (35.18) | 704 (39.42) | 99 (35.74) | 178 (24.76) |
| rIII | 2024 (31.87) | 519 (29.06) | 135 (48.74) | 352 (48.96) |
| Chemotherapy (%) |  |  |  |  |
| No/Unknown | 2734 (43.05) | 689 (38.58) | 180 (64.98) | 293(40.75) |
| Yes | 3617 (56.95) | 1097 (61.42) | 97 (35.02) | 426(59.25) |
| Radiotherapy (%) |  |  |  |  |
| No/Unknown | 3952 (62.23) | 1192 (66.74) | 247 (89.17) | 719(100.0) |
| Yes | 2399 (37.77) | 594 (33.26) | 30 (10.83) | / |
| LNs (median [IQR]) | 16 [10, 24] | 18 [12, 27] | 17 [10, 31] | 35 [26, 45] |
| pLNs (median [IQR]) | 1 [0, 4] | 0 [0, 3] | 2 [0, 7] | 2 [0, 7] |
| LNR (median [IQR]) | 0.019 [0.000, 0.250] | 0.000 [0.000, 0.200] | 0.162 [0.000, 0.500] | 0.053 [0.000, 0.227] |
| Median follow-up (months) | 50 [19, 77] | 30 [20, 37] | 18[11, 28] | 45 [20, 73] |
| 3-Y OS (95%CI) | 60.2(59.0-61.5) | 64.6(62.2-67.1) | 51.2(44.2-59.3) | 81.9(78.8-85.1) |
| 5-Y OS (95%CI) | 49.5(48.2-50.7) | / | 43.3(34.5-54.3) | 74.2(70.5-78.1) |

**Supplementary Table 2.** The rN staging for patients with gastric cancer in the Dataset 1 (Training set).

| LNR-value | Log-rank (Mantel-Cox) | |
| --- | --- | --- |
|  | χ²-value | P-value |
| 0~0.025 | - | - |
| 0.025~0.05 | 5.793 | 0.016 |
| 0.175~0.2 | 4.866 | 0.027 |
| 0.45~0.475 | 5.053 | 0.025 |
| 0.6~0.625 | 6.337 | 0.012 |

**Supplementary Table 3.** Survival comparisons among the 8th AJCC and rAJCC staging system in the Datasets 1,2,3 and 4.

| Stage | AJCC | |  | rAJCC | |
| --- | --- | --- | --- | --- | --- |
|  |  |  |  |  |  |
| Dataset 1 | 5-Y OS (95%CI) | HR |  | 5-Y OS (95%CI) | HR |
| I | 75.6(73.6-77.6) | 1.0(Ref.) | rI | 82.7(81.1-84.3) | 1.0(Ref.) |
| II | 53.9(51.6-56.3) | 1.9 | rII | 63.7(61.8-65.8) | 2.0 |
| III | 28.4(26.7-30.2) | 3.9 | rIII | 32.9(30.9-35.1) | 4.6 |
| Dataset 2 | 3-Y OS (95%CI) | HR |  | 3-Y OS (95%CI) | HR |
| I | 83.5(80.1-87.2) | 1.0(Ref.) | rI | 83.2(79.9-86.6) | 1.0(Ref.) |
| II | 68.2(63.8-72.8) | 2.0 | rII | 67.9(64.1-71.9) | 2.0 |
| III | 50.4(46.7-54.5) | 3.9 | rIII | 40.5(36.1-45.3) | 4.9 |
| Dataset 3 | 5-Y OS (95%CI) | HR |  | 5-Y OS (95%CI) | HR |
| I | 75.8(60.2-95.4) | 1.0(Ref.) | rI | 76.8(61.8-95.4) | 1.0(Ref.) |
| II | 37.0(15.9-86.1) | 1.6 | rII | 46.6(28.2-76.9) | 1.6 |
| III | 34.2(24.7-47.1) | 2.9 | rIII | 29.8(20.1-44.1) | 3.2 |
| Dataset 4 | 5-Y OS (95%CI) | HR |  | 5-Y OS (95%CI) | HR |
| I | 92.1(87.4-97.1) | 1.0(Ref.) | rI | 92.9(88.6-97.4) | 1.0(Ref.) |
| II | 79.1(71.8-87.1) | 2.7 | rII | 80.5(73.7-88.0) | 2.7 |
| III | 63.8(58.4-69.8) | 5.7 | rIII | 60.8(55.0-67.2) | 6.9 |

**Supplementary Table 4.** Discrimination AUC comparison between rAJCC and 8^th^ AJCCstaging system in different datasets

| Dataset 1 | |  |  | Dataset 2 | |  |
| --- | --- | --- | --- | --- | --- | --- |
| months | AUC(AJCC) | AUC(rAJCC) | | months | AUC(AJCC) | AUC(rAJCC) |
| 2.00 | 0.59 | 0.64 |  | 2.00 | 0.58 | 0.63 |
| 6.00 | 0.63 | 0.66 |  | 4.00 | 0.60 | 0.65 |
| 10.00 | 0.66 | 0.69 |  | 6.00 | 0.60 | 0.63 |
| 14.00 | 0.68 | 0.70 |  | 8.00 | 0.62 | 0.65 |
| 18.00 | 0.69 | 0.71 |  | 10.00 | 0.63 | 0.66 |
| 22.00 | 0.70 | 0.72 |  | 12.00 | 0.66 | 0.68 |
| 26.00 | 0.71 | 0.73 |  | 14.00 | 0.65 | 0.68 |
| 30.00 | 0.71 | 0.73 |  | 16.00 | 0.66 | 0.68 |
| 34.00 | 0.71 | 0.73 |  | 18.00 | 0.66 | 0.69 |
| 38.00 | 0.71 | 0.73 |  | 20.00 | 0.66 | 0.69 |
| 42.00 | 0.71 | 0.73 |  | 22.00 | 0.65 | 0.69 |
| 46.00 | 0.71 | 0.73 |  | 24.00 | 0.65 | 0.68 |
| 50.00 | 0.71 | 0.73 |  | 26.00 | 0.66 | 0.69 |
| 54.00 | 0.71 | 0.73 |  | 28.00 | 0.66 | 0.69 |
| 58.00 | 0.72 | 0.73 |  | 30.00 | 0.67 | 0.69 |
| 62.00 | 0.72 | 0.73 |  | 32.00 | 0.66 | 0.69 |
| 66.00 | 0.72 | 0.73 |  | 34.00 | 0.66 | 0.69 |
| 70.00 | 0.71 | 0.73 |  | 36.00 | 0.67 | 0.70 |
| 74.00 | 0.71 | 0.72 |  | 38.00 | 0.68 | 0.71 |
| 78.00 | 0.71 | 0.72 |  | 40.00 | 0.67 | 0.70 |
| 82.00 | 0.72 | 0.73 |  | 42.00 | 0.68 | 0.71 |
| 86.00 | 0.71 | 0.72 |  | 44.00 | 0.67 | 0.71 |
| 90.00 | 0.70 | 0.71 |  | 46.00 | 0.68 | 0.73 |
| 94.00 | 0.69 | 0.70 |  |  |  |  |
| 98.00 | 0.69 | 0.70 |  |  |  |  |
| 102.00 | 0.68 | 0.70 |  |  |  |  |
| 106.00 | 0.67 | 0.68 |  |  |  |  |
| 110.00 | 0.66 | 0.68 |  |  |  |  |
| 114.00 | 0.67 | 0.69 |  |  |  |  |
| 118.00 | 0.63 | 0.66 |  |  |  |  |

| Dataset 3 | |  |  | Dataset 4 | |  |
| --- | --- | --- | --- | --- | --- | --- |
| months | AUC(AJCC) | AUC(rAJCC) | | months | AUC(AJCC) | AUC(rAJCC) |
| 1.50 | 0.75 | 0.76 |  | 2.10 | 0.54 | 0.57 |
| 5.50 | 0.59 | 0.53 |  | 6.10 | 0.63 | 0.66 |
| 9.50 | 0.61 | 0.59 |  | 10.10 | 0.69 | 0.71 |
| 13.50 | 0.62 | 0.64 |  | 14.10 | 0.66 | 0.66 |
| 17.50 | 0.61 | 0.63 |  | 18.10 | 0.65 | 0.67 |
| 21.50 | 0.61 | 0.64 |  | 22.10 | 0.65 | 0.67 |
| 25.50 | 0.58 | 0.61 |  | 26.10 | 0.66 | 0.68 |
| 29.50 | 0.64 | 0.67 |  | 30.10 | 0.66 | 0.69 |
| 33.50 | 0.65 | 0.67 |  | 34.10 | 0.67 | 0.70 |
| 37.50 | 0.59 | 0.62 |  | 38.10 | 0.67 | 0.71 |
| 41.50 | 0.60 | 0.67 |  | 42.10 | 0.68 | 0.71 |
| 45.50 | 0.66 | 0.74 |  | 46.10 | 0.68 | 0.71 |
| 49.50 | 0.68 | 0.74 |  | 50.10 | 0.68 | 0.71 |
| 53.50 | 0.67 | 0.72 |  | 54.10 | 0.68 | 0.71 |
| 57.50 | 0.66 | 0.70 |  | 58.10 | 0.68 | 0.70 |
| 61.50 | 0.62 | 0.65 |  | 62.10 | 0.67 | 0.70 |
| 65.50 | 0.61 | 0.61 |  | 66.10 | 0.67 | 0.71 |
| 69.50 | 0.57 | 0.57 |  | 70.10 | 0.67 | 0.71 |
| 73.50 | 0.58 | 0.58 |  | 74.10 | 0.68 | 0.70 |
| 77.50 | 0.58 | 0.58 |  | 78.10 | 0.68 | 0.72 |
| 81.50 | 0.55 | 0.55 |  | 82.10 | 0.70 | 0.73 |
| 85.50 | 0.50 | 0.50 |  | 86.10 | 0.69 | 0.73 |
| 89.50 | 0.50 | 0.50 |  | 90.10 | 0.71 | 0.75 |
| 93.50 | 0.50 | 0.50 |  | 94.10 | 0.74 | 0.78 |
| 97.50 | 0.50 | 0.50 |  | 98.10 | 0.74 | 0.78 |
|  |  |  |  | 102.10 | 0.74 | 0.78 |
|  |  |  |  | 106.10 | 0.74 | 0.78 |
|  |  |  |  | 110.10 | 0.74 | 0.79 |
|  |  |  |  | 114.10 | 0.72 | 0.76 |
|  |  |  |  | 118.10 | 0.71 | 0.76 |

**Supplementary Table 5.** Survival comparisons among the 8th AJCC and rAJCC staging systems in patient with ELN count≥30.

| Stage | AJCC stage | |  | rAJCC stage | |
| --- | --- | --- | --- | --- | --- |
|  |  |  |  |  |  |
| Internal validation set-1 | 5-Y OS(95%CI) | HR |  | 5-Y OS(95%CI) | HR |
| I | 85.3(80.6-90.3) | 1.0(Ref.) | rI | 82.0(77.2-87.0) | 1.0(Ref.) |
| II | 66.3(60.4-72.7) | 2.2 | rII | 59.6(54.8-64.7) | 2.4 |
| III | 35.8(31.8-40.2) | 5.3 | rIII | 26.6(22.2-32.0) | 6.0 |
| Internal validation set-2 | 3-Y OS(95%CI) | HR |  | 3-Y OS(95%CI) | HR |
| I | 88.7(80.9-97.2) | 1.0(Ref.) | rI | 86.9(79.8-94.6) | 1.0(Ref.) |
| II | 74.2(66.0-83.4) | 2.3 | rII | 72.2(65.2-80.0) | 1.9 |
| III | 56.0(48.7-64.3) | 4.6 | rIII | 44.7(35.6-56.3) | 5.1 |
| External validation set-1 | 3-Y OS(95%CI) | HR |  | 3-Y OS(95%CI) | HR |
| I | 90.0(73.2-100.0) | 1.0(Ref.) | rI | 90.9(75.4-100.0) | 1.0(Ref.) |
| II | 62.3(34.1-100.0) | 2.8 | rII | 74.6(53.0-100.0) | 1.9 |
| III | 49.7(35.4-69.8) | 5.9 | rIII | 33.9(19.5-58.9) | 10.3 |
| External validation set-2 | 5-Y OS(95%CI) | HR |  | 5-Y OS(95%CI) | HR |
| I | 93.4(87.7-99.5) | 1.0(Ref.) | rI | 96.5(93.2-99.9) | 1.0(Ref.) |
| II | 84.5(76.6-93.2) | 3.1 | rII | 93.7(88.9-98.7) | 2.6 |
| III | 67.8(61.2-75.2) | 8.1 | rIII | 73.4(67.1-80.2) | 8.9 |
